# Declining coral calcification to enhance twenty-first-century ocean carbon uptake by gigatonnes

**DOI:** 10.1073/pnas.2501562122

**Published:** 2025-06-02

**Authors:** Lester Kwiatkowski, Alban Planchat, Marc Pyolle, Olivier Torres, Nathaelle Bouttes, Adrien Comte, Laurent Bopp

**Affiliations:** ^a^Laboratoire d’océanographie et du climat: expérimentations et approches numériques, Institut Pierre-Simon Laplace, Sorbonne Université, CNRS, Institut de recherche pour le développement, Muséum national d’Histoire naturelle, Paris 75005, France; ^b^Laboratoire de Météorologie Dynamique, Institut Pierre-Simon Laplace, Ecole Normale Supérieure/Université Paris Sciences et Lettres, Département de Géosciences, CNRS, Ecole Polytechnique, Sorbonne Université, Paris 75005, France; ^c^Laboratoire des Sciences du Climat et de l’Environnement, Institut Pierre-Simon Laplace, Commissariat à l’Énergie Atomique et aux Énergies Alternatives, CNRS, Université Paris-Saclay, Gif-sur-Yvette 91191, France; ^d^Laboratoire des sciences de l’environnement marin, Institut de recherche pour le développement, Université Brest, CNRS, Ifremer, Plouzané 29280, France

**Keywords:** coral reef, carbon, climate, feedback, calcification

## Abstract

The sensitivity of coral reefs to climate change is well established. As the oceans warm and acidify, the calcification of coral reefs declines with net calcium carbonate dissolution projected under even moderate emissions trajectories. The impact of this on the global carbon cycle is however yet to be accounted for. Here, we use a synthesis of the sensitivity of coral reef calcification to climate change, alongside reef distribution products to estimate alkalinity and dissolved inorganic carbon fluxes resulting from reductions in reef calcification. Using a global ocean biogeochemical model, we simulate the impact on ocean carbon uptake under different emissions scenarios, accounting for uncertainty in present-day calcification rates. Reductions in net coral reef carbonate production can enhance the ocean carbon sink by up to 1.25 GtCO_2_ y^−1^ by midcentury (0.48 GtCO_2_ y^−1^ median estimate) with cumulative ocean carbon uptake up to 13% greater by 2300 (7% median estimate). Our findings indicate that accounting for the coral reef feedback in projections will increase estimates of the remaining carbon budget associated with global warming thresholds, as well as the likelihood that net zero emissions can be achieved without negative emissions.

## Significance

Climate change is anticipated to suppress coral reef calcification with net dissolution expected under even moderate emissions scenarios. The impact of this on the global carbon cycle is however unknown as these ecosystems are not represented in Earth system models. Using a global biogeochemical model, we estimate that the coral reef climate feedback will enhance ocean carbon uptake by up to 13% by the year 2300. This negative feedback increases estimates of the remaining carbon budget associated with global warming thresholds such as the Paris Agreement goal of limiting the global increase in temperature to 2 °C above preindustrial levels.

The oceans currently absorb around 25% of anthropogenic CO_2_ emissions (1, 2), limiting the fraction that remains in the atmosphere and the resulting climate change. Future ocean carbon uptake is therefore a key determinant of the remaining carbon budgets associated with global warming targets. The solubility pump is the principal driver of projected ocean carbon uptake (1) and is mainly driven by the CO_2_ partial pressure (*p*CO_2_) gradient between the atmosphere and surface ocean. While the projected change in the air–sea *p*CO_2_ gradient, and therefore future ocean carbon uptake, is dominated by anthropogenic emissions of CO_2_ (3–5), perturbations to ocean biogeochemical processes (6–8) and model uncertainty (2, 4) can also affect uptake.

Coral reef ecosystems have long been known to modify local seawater chemistry and in turn air–sea CO_2_ fluxes (9–11). The dominant processes responsible for such changes are net ecosystem production (gross primary production minus respiration; NEP) and net ecosystem calcification (gross calcification minus CaCO_3_ dissolution; NEC). Positive net production reduces dissolved inorganic carbon (DIC) concentrations, decreasing seawater *p*CO_2_. Positive net calcification however, reduces total alkalinity (Alk) and DIC, in a ratio of 2:1, increasing the *p*CO_2_ of seawater. The balance of organic-to-inorganic carbon production is therefore the principal driver of whether a reef acts as a net source or sink of atmospheric CO_2_. This balance can be influenced by multiple factors including community composition (12, 13), nutrient supply (14), disease (15) and grazing pressure (16). Historically however, studies have shown that the relatively low organic production and high calcification of coral reefs produces a net source of CO_2_ to the atmosphere (17–19).

Alongside other stressors, ocean warming and acidification act to reduce the net ecosystem calcification of coral reefs through processes such as bleaching (20–22), reduced calcification (23, 24), and enhanced sediment dissolution (25). Observations suggest that global net calcification is already regionally declining (23, 26, 27), with models projecting a midcentury transition to a state of net CaCO_3_ dissolution (negative NEC) under even moderate emissions scenarios (25, 28–30). Other factors being equal, this decline will increase ocean alkalinity and DIC in a 2:1 molar ratio that acts to reduce the *p*CO_2_ of seawater and enhance ocean carbon uptake from the atmosphere. It therefore represents a negative climate feedback currently unresolved by Earth system models (31) and may contribute to biases in projected ocean carbon uptake (4).

## Simulating Declining Coral Reef Calcification

**Fig. 1.**
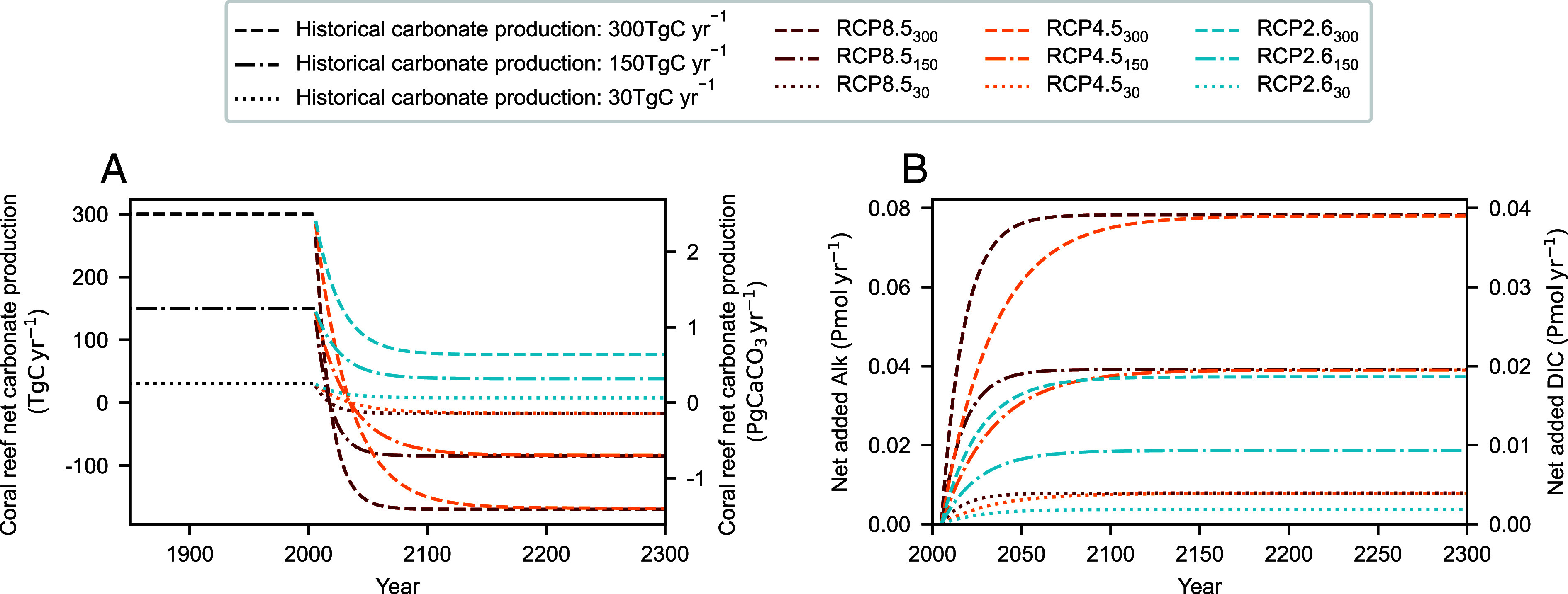
Global declines in coral reef carbonate production and the associated fluxes of alkalinity and DIC. The (*A*), estimated global coral reef net ecosystem calcification (TgC y^−1^ and PgCaCO_3_ y^−1^) and the (*B*), simulated global flux of alkalinity (Pmol y^−1^) and DIC associated with carbonate production decline in each RCP. Black lines represent the historical unperturbed rate of global carbonate production (30, 150, and 300 TgC y^−1^), with the same line type used for corresponding RCP simulations and the assumed carbonate historical production rate denoted in subscript.

We simulate the biogeochemical impact of declining coral reef net calcification as an anomalous source of alkalinity and DIC in ocean regions containing reefs. The magnitude of these fluxes is dependent on both the assumed historical rate of global coral reef calcification and the decline under different future emissions scenarios (Fig. 1). We derived nine time-evolving estimates of future alkalinity and DIC fluxes, based on three historical values of global coral reef calcification (30, 150, and 300 TgC y^−1^) thought to encompass observational uncertainty (32), and exponential decay functions of calcification under different emissions scenarios (Representative Concentration Pathways 2.6 (RCP2.6), 4.5 (RCP4.5), and 8.5 (RCP8.5) (33). Our central historical calcification estimate of 150 TgC y^−1^ is consistent with previously published best estimates and a likely range of 30 to 300 TgC y^−1^ (32, 34). The functions of declining net calcification are based on a meta-analysis of experimental, mesocosm, and in situ estimates of coral reef calcification under each RCP, and include both living and nonliving components of the reef carbonate budget (e.g. coral and crustose coralline algae calcification, sediment dissolution, and bioerosion) (28). The anomalies in carbonate production are estimated to year 2300 assuming an inexhaustible CaCO_3_ reservoir. Given the current reef CaCO_3_ reservoir has typically arisen over the last 5000 to 10,000 y and maximal net dissolution rates are less than historical calcification rates, this assumption is supported on multicentennial timescales.

The impact of the coral reef derived fluxes of alkalinity and DIC on ocean anthropogenic carbon uptake (C_ant_) was simulated using the PISCES global ocean biogeochemical model (35). PISCES simulates the cycles of organic and inorganic ocean carbon, total alkalinity, oxygen, and essential marine nutrients (N, P, Si, and Fe). It includes two phytoplankton functional types and two zooplankton size classes, alongside two particulate organic matter size classes, particulate inorganic matter (calcite) and dissolved organic matter. Pelagic calcification is implicitly parameterized as a function of organic matter production and consistent with current generation ocean biogeochemical models. However sediment dissolution and benthic calcification, including that of coral reefs, are unresolved (31). Air–sea CO_2_ fluxes, follow Ocean Model Intercomparison Project protocols (36), with gas exchange dependent on the air–sea partial pressure gradient and a parameterization of the instantaneous gas transfer velocity that depends on 10 m atmospheric wind speed (37, 38).

This PISCES version utilized is similar to that described by Aumont et al. (2015) (35) and used in multiple Earth system models (e.g. IPSL-CM6A-LR) (39), however the burial fraction of plankton-derived particulate inorganic carbon was adjusted so that the global alkalinity inventory is conserved at preindustrial levels without the need of a restoring scheme (40) and the parameterization of diazotrophy was modified (41). The model was forced with physical ocean outputs derived from the IPSL-CM5A-LR Earth system model and concentrations of greenhouse gases consistent with the RCPs (33). Coral reef derived fluxes of alkalinity and DIC were simulated as boundary conditions in the surface ocean of regions where reefs have been identified (42), in a molar ratio of 2:1 consistent with a decline in net carbonate production. These fluxes are provided continuously and neglect subannual and regional diversity in the rate of decline.

## Ocean Carbon Sink Enhancement

**Fig. 2.**
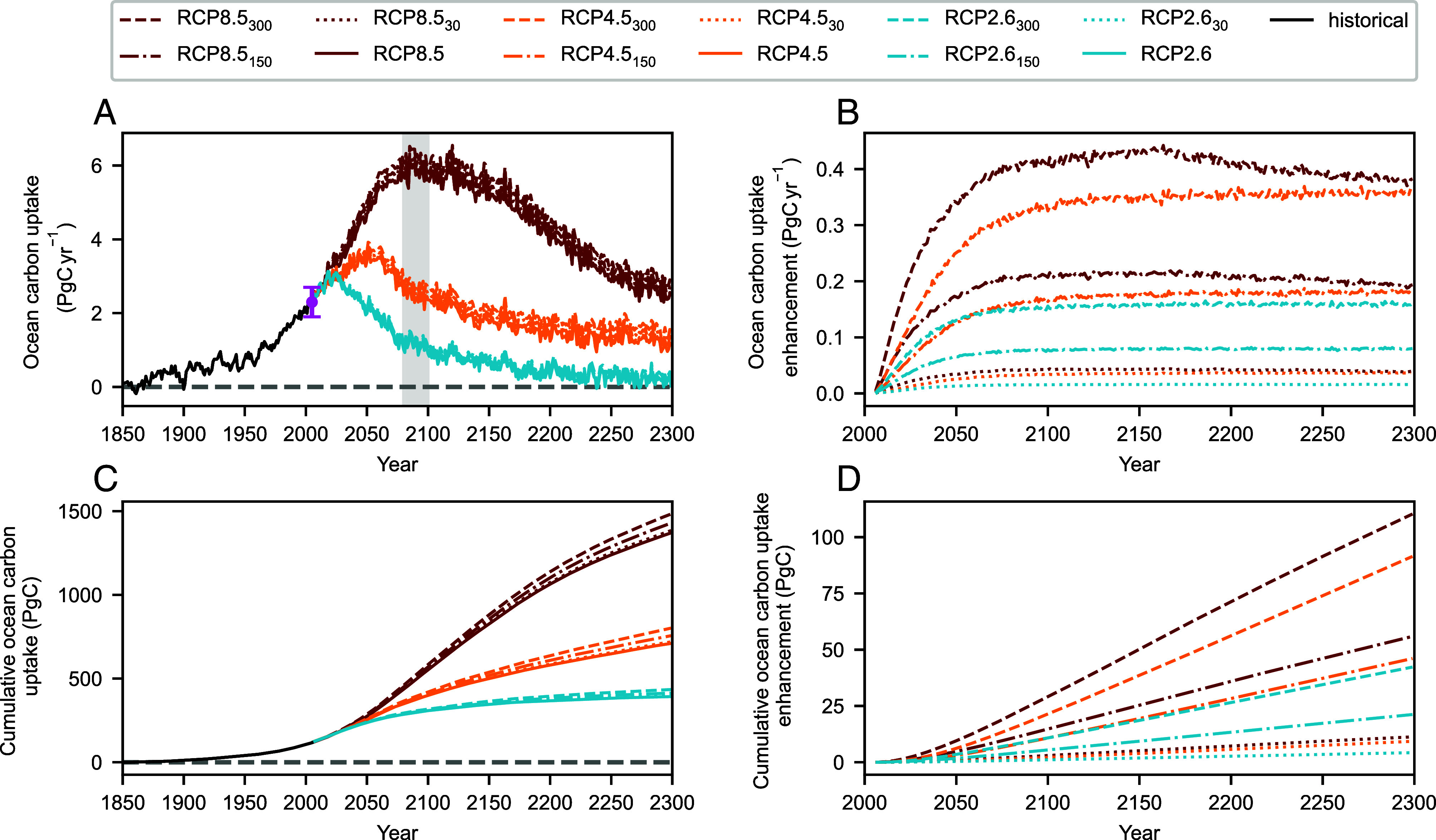
Enhancement of the global ocean anthropogenic carbon sink due to reductions in coral reef carbonate production. The simulated (*A*), annual ocean anthropogenic carbon uptake (PgC y^−1^), (*B*), enhancement due to coral reef degradation (PgC y^−1^), (*C*), cumulative anthropogenic ocean carbon uptake (PgC) and (*D*), enhancement due to coral reef degradation (PgC). Anomalies in ocean carbon uptake due to coral reef carbonate production declines are shown relative to their respective RCP. No decline in coral reef carbonate production is simulated prior to 2005. Data-based estimates of the ocean anthropogenic carbon sink in the 2000s are shown in magenta in panel A with error bars representing 1 SD (2). The gray bar in panel A corresponds to the period shown in Fig. 3.

Our historical simulation of ocean anthropogenic carbon uptake is broadly consistent with the magnitude and spatial distribution of observational estimates (Fig. 2 and *SI Appendix*, Fig. S1). In future scenarios, the principal driver of divergence in projected anthropogenic carbon uptake by the ocean is the atmospheric CO_2_ concentration associated with each RCP (Fig. 2). In the absence of coral reefs, the C_ant_ flux peaks at 3.0, 3.7, and 6.1 PgC y^−1^ in RCP2.6, RCP4.5, and RCP8.5, respectively. This is consistent with ESM simulations performed as part of the CMIP exercises (4, 5). The coral reef carbonate feedback can however substantially enhance projected C_ant_ uptake.

Coral-driven increases in the ocean carbon sink reach near-maximal values in the mid 21st century, with sink enhancement maintained on multicentennial timescales. Under the median estimate of historical reef carbonate production combined with the moderate emissions scenario (RCP4.5_150_), C_ant_ uptake is enhanced by 0.13 PgC y^−1^ by 2050, increasing to 0.17 PgC y^−1^ in 2100 and persisting at this level until 2300 (Fig. 2). The range in peak C_ant_ uptake enhancement across the simulation ensemble ranges from 0.02 PgC y^−1^ (RCP2.6_30_ in year 2211) to 0.44 PgC y^−1^ (RCP8.5_300_ in year 2163). In the median scenario (RCP4.5_150_), the cumulative ocean carbon sink is enhanced by 11 PgC by 2100 (3%), with this increasing to 46 PgC by 2300 (7%) and by up to 110 PgC (8%) in 2300 under RCP8.5_300_. Given that the end-of-century range in ESM estimates of ocean carbon uptake is between ~0.2 PgC y^−1^ (for the highest mitigation scenarios) and ~0.8 PgC y^−1^ (for highest emission scenarios) (4), coral reef induced enhancement of the ocean carbon sink represents a relatively substantial potential contributor to model uncertainty that is currently unaccounted for.

Uncertainty in the coral reef induced enhancement of ocean C_ant_ uptake is primarily a consequence of the assumed historical carbonate production rate and, to a lesser extent, scenario uncertainty (RCP). The C_ant_ uptake enhancement is similar between RCP4.5 and RCP8.5 simulations for a given historical carbonate production rate, indicative of maximum carbonate production declines being reached under moderate emissions scenarios (*Materials and Methods*). This also reflects the characterization of coral reef ecosystems as potential tipping elements in the Earth system that are susceptible to abrupt change even at current warming levels (43). Refining estimates of both historical coral reef carbonate production as well as its sensitivity to climate change is therefore critical to constraining estimates of the enhancement of ocean carbon uptake.

**Fig. 3.**
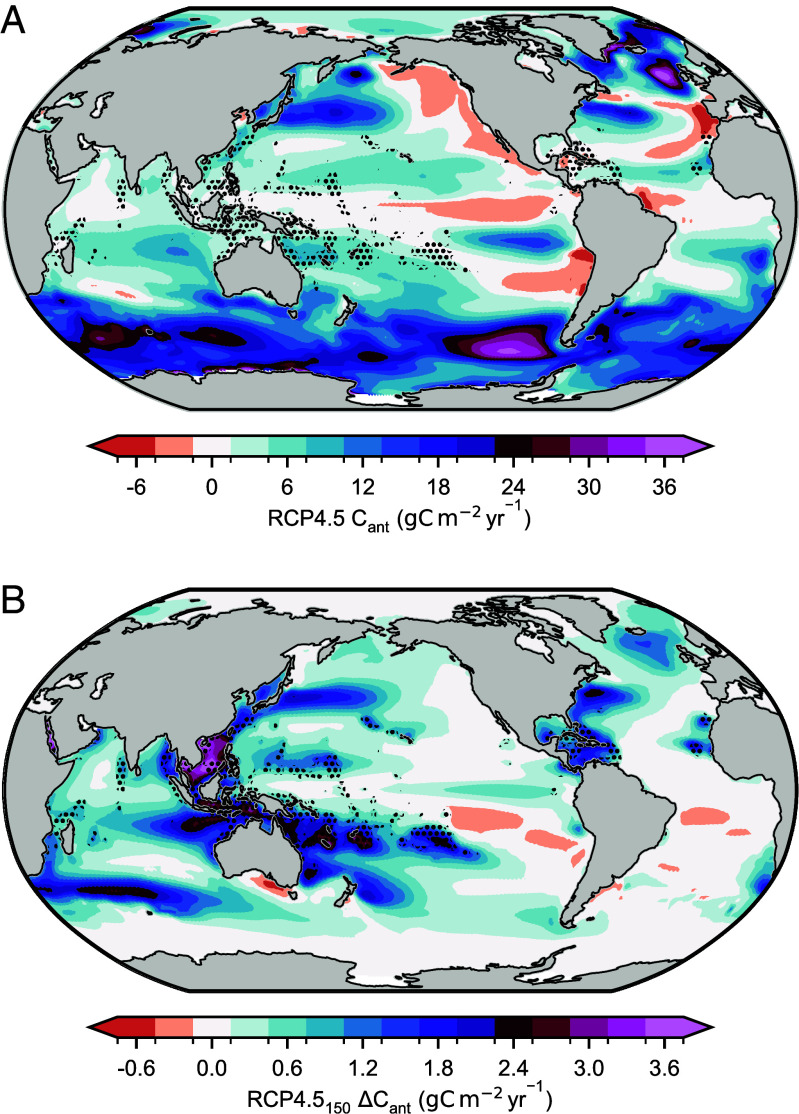
Carbon uptake enhancement extends beyond the regions of coral reefs. The (*A*), mean ocean anthropogenic carbon uptake in 2081 to 2099 of RCP4.5 and the (*B*), coral reef-driven anomaly in ocean anthropogenic carbon uptake in 2081 to 2099 of RCP4.5_150_. Stippling indicates the distribution of coral reefs.

The simulated increase in ocean anthropogenic carbon uptake is locally highest in the regions of coral reefs such as the coral triangle (Fig. 3). However enhanced uptake outside coral regions is responsible for 50 to 80% of the global enhancement (*SI Appendix*, Fig. S2). This is indicative of the seawater residence time in reef regions being less than the air–sea equilibration time of induced *p*CO_2_ anomalies and has been similarly demonstrated in simulations of afforestation and alkalinity enhancement (44).

The fraction of enhancement that occurs in reef regions is almost entirely determined by the atmospheric CO_2_ concentration (i.e. RCP) and not the historical global carbonate production rate (*SI Appendix*, Fig. S2). This is indicative of the balance between seawater residence time and air–sea equilibration time exhibiting limited change for different reef net calcification anomalies in our simulations. While most of the ocean carbon uptake enhancement is located in reef regions when reef calcification initially decreases (*SI Appendix*, Fig. S2), the fraction occurring in coral regions then declines across the three RCPs to around 25% in 2040. This reflects increasing advection of unequilibrated water masses out of coral regions and limited differences between scenarios up to this point. In RCP2.6, the fraction further declines to around 18% in 2300 while in RCP4.5 the decline is more gradual to around 23% in 2300. Counterintuitively, under RCP8.5 the fraction increases around 2070 to ~50% of the global enhancement in 2300. This is likely due to the reduction in CO_2_ equilibration time in this very high CO_2_ scenario (45) alongside increases in surface seawater residence time.

## Carbon Budget Consequences

Our simulations indicate that the coral reef feedback could increase 21st century ocean carbon uptake by up to 5%. This effect is smaller than that of the soft tissue pump, which enhances ocean carbon uptake by 5 to 17% in moderate-to-high emissions scenarios, according to current ESMs (46). Notably, the soft tissue pump achieves this increase despite a global decline in organic carbon export. Instead, the enhancement results from longer residence times of respired DIC due to increased stratification and a slowdown in ocean overturning. In the absence of circulation changes, pelagic carbonate pump changes have been estimated to increase 21st century ocean carbon uptake by up to 5% in ESMs however this is highly uncertain with reductions in carbon uptake also simulated (40).

The identified coral reef feedback, currently unresolved in ESMs, increases remaining carbon budgets associated with given temperature thresholds and therefore has policy consequences. The latest IPCC assessment report provides an upper estimate and likelihood associated with currently unresolved Earth system feedbacks (e.g. permafrost melt) (1). Based on our simulations the coral reef feedback has a potential 21st century magnitude comparable to boreal forest dieback (although opposite in sign). This is substantially larger than other potential feedbacks due to unresolved marine ecology in ESMs. For example, the impact of climate change on planktonic ecosystem structure and elemental stoichiometry is estimated to decrease ocean carbon uptake by < 1% (8), while climate-driven proliferation of gelatinous zooplankton has been shown to enhance ocean carbon uptake by < 0.1% (47). Moreover, contrary to most documented unresolved Earth system feedbacks, the likelihood of the coral reef feedback occurring this century is very high. Indeed, multiple lines of evidence indicate it is probably already occurring (23, 27).

Scenarios consistent with the Paris Agreement goal of limiting the global increase in temperature to 2 °C above preindustrial levels have associated cumulative negative emissions of 330 GtCO_2_ (260-440 GtCO_2_ interquartile range) (48). However cumulative ocean carbon uptake over the 21st century may be underestimated by up to 11 GtCO_2_ in high-mitigation scenarios consistent with this goal due to the coral reef feedback (RCP2.6_300_), with a corresponding decrease in required negative emissions.

The global surface air temperature change associated with net zero emissions, also known as the zero emissions commitment (ZEC), is estimated between −0.36 and 0.29 °C after 50 y of ceased emissions (49). This range reflects uncertainty in whether the stabilization of global temperatures is consistent with limited residual emissions or will indeed require sustained negative emissions. Ocean carbon uptake post zero emissions is a critical determinant of the ZEC, with greater uptake offsetting declines in ocean heat uptake and therefore typically associated with a lower ZEC (49). Estimates of ZEC however, are derived from models that fail to account for the coral reef feedback. Its inclusion would enhance ocean carbon uptake post zero emissions, reducing the ZEC and therefore increasing the likelihood that stabilizing global temperature can be achieved without negative emissions.

## Coral Reef Organic Production and Other Assumptions

In contrast to net calcification, coral reef organic net ecosystem production is assumed constant in our simulations. This has the potential to influence the magnitude of the simulated coral reef feedback. A general increase in coral reef organic production coincident with calcification declines over the last 50 y has been documented (50). Such increases would typically act to increase the projected coral reef-induced enhancement of the ocean carbon sink. Nonetheless, local studies have documented declines in net organic production post bleaching and suggested such trends may continue under future warming (51–53). This would likely act to attenuate the projected enhancement of the ocean carbon sink. Some reefs have even been shown to be in a current state of both net carbonate dissolution and net respiration, with each metabolic cycle highly seasonally variable and future changes undetermined (54). In short, the response of coral reef organic production to climate change is highly uncertain, likely to be spatially variable and could act to both increase and attenuate the projected enhancement of the ocean carbon sink. We currently lack the observational and experimental constraints required to simulate transient changes in reef organic production but, when possible, this should be incorporated into projections to refine estimates of the coral reef feedback.

Our simulations assume no spatial heterogeneity in reef-derived inputs of alkalinity and DIC in response to carbonate production declines. This is inconsistent with observations which show spatial variability in both present-day coral net carbonate production rates and estimated declines in response to climate change (28, 50, 55). Many Caribbean reefs are already at accretionary stasis or net dissolving (54, 55) while Pacific reefs generally exhibit higher present-day carbonate production and are considered more likely to sustain positive net carbonate production in the future (28). Incorporating such heterogeneity into projections may therefore affect regional carbon uptake enhancement. However given that the sensitivity of ocean carbon uptake to alkalinity increases exhibits limited spatial variability across the tropics (56), the impact on global ocean carbon uptake is likely to be minimal. Within this context, it should be noted that the impact of other stressors such as fishing pressure, increased disease prevalence, cyclone intensity, and nutrification (29, 57, 58) is currently unaccounted for in global estimates of future coral carbonate production (28, 59). Incorporating these stressors into projections is likely to enhance projected declines in calcification, increase ocean carbon sink enhancement and introduce additional spatiotemporal variability.

A further limitation of our simulations is that atmospheric CO_2_ concentrations are prescribed and therefore unaffected by the enhanced air–sea carbon fluxes attributable to reductions in coral reef carbonate production. In an emissions-driven model, enhanced ocean carbon uptake would act to decrease atmospheric CO_2_ concentrations, typically reducing terrestrial carbon uptake and resulting in lesser overall enhancement of the ocean carbon sink (60). Assessing the magnitude of the coral reef carbonate pump feedback in simulations that account for atmospheric and terrestrial carbon cycle feedbacks is therefore required. The recent development of an Earth system model of intermediate complexity coral reef module (61), which might be adapted for implementation within ESMs, represents progress in this direction.

## Measuring the Feedback in the Real World

Confirmation of the coral reef feedback with measurements is clearly desirable. Coral reef alkalinity measurements are perhaps most promising in this regard as they are both conservative with respect to temperature variations and unaffected by air–sea gas exchange. However, the maximum simulated change in surface ocean alkalinity induced by carbonate production declines is approximately 50 mmol m^−3^ century^−1^ in Indian Ocean regions of RCP8.5_300_ (*SI Appendix*, Fig. S3). In our median scenario (RCP4.5_150_) the simulated surface ocean alkalinity trend is reduced by half, with most coral reef regions exhibiting increases of 10 to 20 mmol m^−3^ century^−1^. Although trends in alkalinity would be higher at the spatial scales of an actual reef, the extremely high natural variability of alkalinity in reef environments (62) (*SI Appendix*, Fig. S3) suggests that multidecadal to multicentennial time series are required for the impact of declining reef calcification on seawater carbonate chemistry to emerge from natural variability. For this reason, the best estimates of the future coral reef climate feedback may depend on alternative, more indirect estimates of changing reef carbonate budgets (e.g. refs. 31 and 32).

## Materials and Methods

### Model and Simulations.

As the biogeochemical models developed for ESMs lack a representation of coral reefs (31), we impose the flux of alkalinity and DIC that results from decreasing coral reef carbonate production as a time-dependent model boundary condition. This perturbation approach is possible because a given decline in net ecosystem calcification has an identical impact on seawater alkalinity and DIC regardless of whether net ecosystem calcification remains positive or transitions to a negative state (i.e. net dissolution). The addition of alkalinity and DIC in coral reef regions is based on published central estimates of present-day reef carbonate production, and production declines under RCP2.6, RCP4.5, and RCP8.5 (28). The central estimate of present-day coral reef carbonate production is 2.8 kgCaCO_3_ m^−2^ y^−1^. This has been projected to decrease to 0.95, −0.63, and −1.45 kgCaCO_3_ m^−2^ y^−1^ in 2050 and thereafter to 0.73, −1.39, and −1.57 kgCaCO_3_ m^−2^ y^−1^ in 2100 under RCP2.6, RCP4.5, and RCP8.5, respectively due to warming and acidification (28). These projections do not account for other stressors such as fishing pressure, greater disease exposure, and nutrification which would likely exacerbate declines (27, 29, 57, 58). We do not account for the uncertain potential of symbiont adaptive capacity to influence projections of future coral reef carbonate production (59). Under low emissions scenarios, such adaptive capacity may result in less severe production declines, while under medium and high emissions, the transition to net dissolution could be delayed. Although the RCPs have been updated to Shared Socioeconomic Pathways (SSPs) in the latest coupled model intercomparison project (63), each of the RCPs used here has an SSP analogue that exhibits similar—although generally greater—surface ocean warming and acidification for a comparable radiative forcing (64).

By fitting exponential decay functions to these estimates, relative anomalies in future reef carbonate production can be estimated annually. However, to convert these into fluxes of alkalinity and DIC, the historical (pre-2005) global carbonate production of coral reefs is required. We adopt a central estimate of historical global carbonate production of 150 TgC y^−1^ (1.25 PgCaCO_3_ y^−1^). This is consistent with previously published central estimates with a likely range of 30 to 300 TgC y^−1^ (32) and global reef carbonate production estimates (78 to 100 TgC y^−1^) based on lower historical estimates of reef extent (34).

In order to encompass the uncertainty in historical global carbonate production we perform simulations for each RCP assuming carbonate production of 30, 150, and 300 TgC y^−1^ (0.25, 1.25, and 2.50 PgCaCO_3_ y^−1^). These carbonate production values are denoted as subscripts in simulation names (i.e. the nine future simulations are RCP2.6_30_, RCP2.6_150_, RCP2.6_300_, RCP4.5_30_, RCP4.5_150_, RCP4.5_300_, RCP8.5_30_, RCP8.5_150_, RCP8.5_300_). In addition to these simulations, an 1850 to 2005 historical simulation is performed, from which the nine RCP simulations are extended in 2005 to 2300. A coincident preindustrial control simulation is also conducted to remove model drift and calculate anthropogenic carbon fluxes. We assume that the impact of reductions in coral reef carbonate production starts to occur in 2005, coincident with the RCPs. This is inconsistent with at least regional evidence that reef carbonate production may have declined prior to 2005 (23, 26, 27) but we lack the data to constrain global production declines over the 1850 to 2005 historical simulations (28). Any such decline however, would act to increase the magnitude of the simulated ocean carbon uptake enhancement over the duration of our simulations.

In the simulation of minimal carbonate production decline (RCP2.6_30_), reef-derived alkalinity and DIC fluxes increase to 0.004 and 0.002 Pmol globally by 2100, respectively, where they are maintained until 2300. While in the simulation of maximal carbonate production decline (RCP8.5_300_), alkalinity and DIC fluxes increase to 0.08 and 0.04 Pmol by 2100, respectively, where thereafter they are maintained. Reef-derived model inputs of alkalinity and DIC are equally distributed across all model grid cells documented to contain warm water coral reefs. As such, we do not account for any regional disparities in the resilience of coral reef carbonate production. We also do not account for the potential for carbonate production declines to reduce projected ocean acidification and therefore attenuate acidification-driven declines in carbonate production. However, the impact of carbonate production declines on projected ocean acidification is highly limited (*SI Appendix*, Fig. S4). Across all simulations most of the decline in coral reef carbonate production occurs prior to 2050 with limited declines thereafter and virtually no further decline post 2150. This is consistent with metanalysis studies (28) and evidence that coral reefs represent a tipping element susceptible to abrupt change at current warming levels (43).

### Model Evaluation.

The PISCES model utilized here is similar to that in multiple ESMs that have contributed to the latest coupled model intercomparison project phase 6 (CMIP6). Our offline model configuration has previously been used to assess potential evolution of the pelagic carbonate pump and the simulated historical anthropogenic carbon uptake in the absence of the coral reef feedback, and is consistent with data-based ocean carbon uptake estimates (2) (*SI Appendix*, Fig. S1) and the CMIP6 ensemble spread (40). Both the spatial distribution of simulated air–sea total carbon fluxes and recent historical trends in globally integrated fluxes are consistent across model simulations and observational products.

## Supplementary Material

Appendix 01 (PDF)

## Data Availability

Model configuration files have been deposited in Zenodo (https://doi.org/10.5281/zenodo.8421951) (65).
