# Supplementary material for: Declining coral calcification to enhance twenty-first-century ocean carbon uptake by gigatonnes
Source: Proc Natl Acad Sci U S A. 2025 Jun 2;122(23):e2501562122. doi: 10.1073/pnas.2501562122 (PMC12167964; doi:10.1073/pnas.2501562122)
Supplement: Supplementary file 1 — Appendix 01 (PDF) [file pnas.2501562122.sapp.pdf]

PNAS

Supporting Information for

Declining coral calcification to enhance twenty-first century ocean carbon uptake by gigatonnes

Lester Kwiatkowski, Alban Planchat, Marc Pyolle, Olivier Torres, Nathaelle Bouttes, Adrien Comte, Laurent Bopp

Corresponding author: Lester Kwiatkowski
Email: lester.morgan-kwiatkowski@locean.ipsl.fr

This PDF file includes:

Figures S1 to S4
SI References

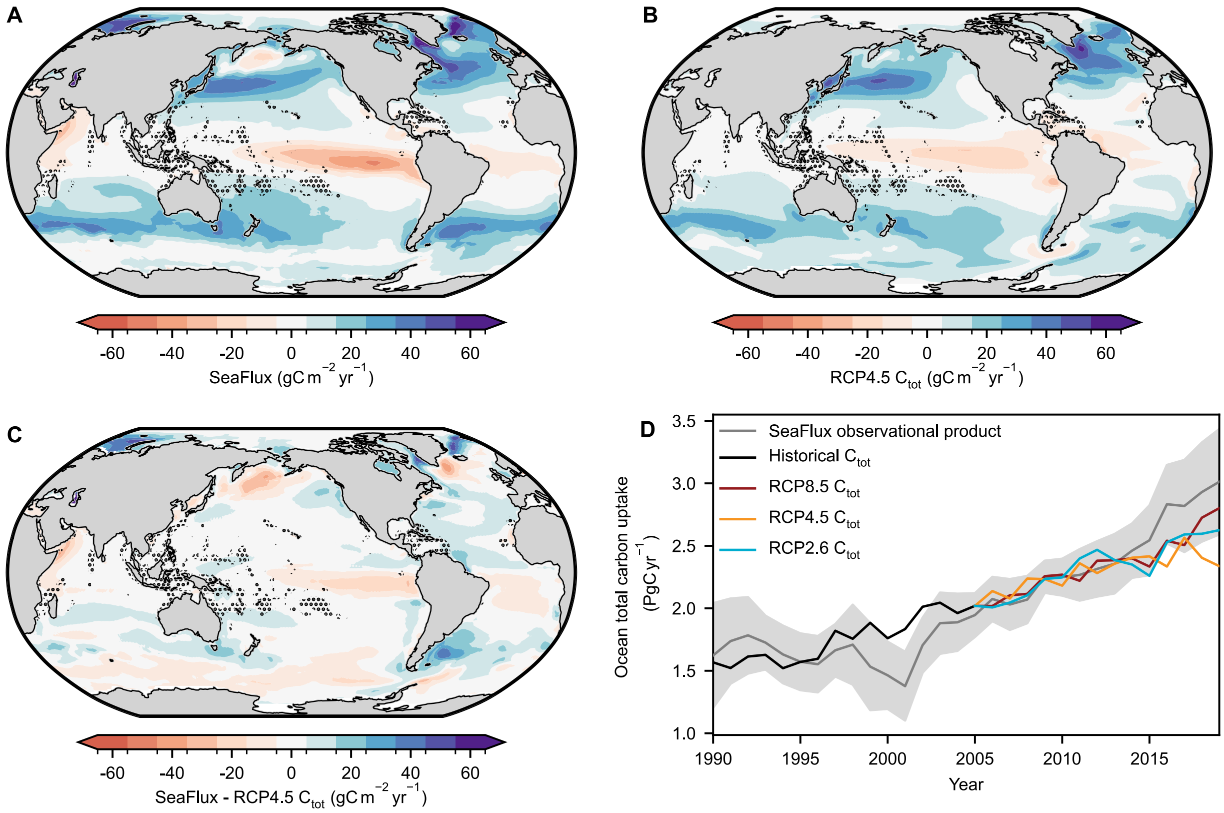

Fig S1. Comparison of simulated ocean carbon uptake with observational products. The (**A**), SeaFlux mean observational product of ocean total carbon flux in 2012-2021 (1), the (**B**), corresponding values in the NEMO-PISCES simulation of RCP4.5 (in the absence of the representation of coral reefs) and the (**C**), simulation anomalies relative to SeaFlux. Stippling indicates the distribution of coral reefs. (**D**), the globally integrated observed and simulated total carbon fluxes from 1990 to 2021. All fluxes are positive into the ocean.

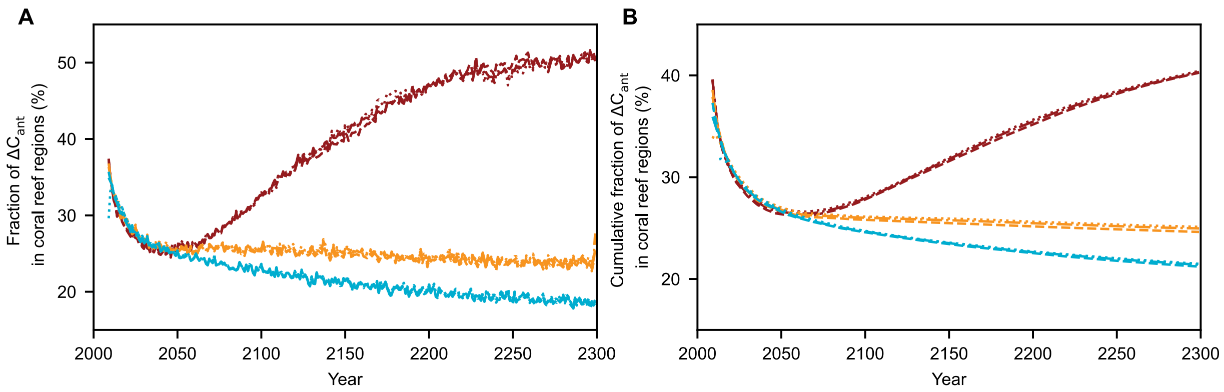

Fig. S2. The spatial distribution of ocean carbon uptake enhancement depends on emission scenario. The fraction of the (**A**), annual and (**B**), cumulative global enhancement in ocean anthropogenic carbon uptake that occurs in coral reef regions. The simulation legend is the same as in Figure 1. The first 4-8 years of each RCP are not shown due to the initially high sensitivity of relative anomalies.

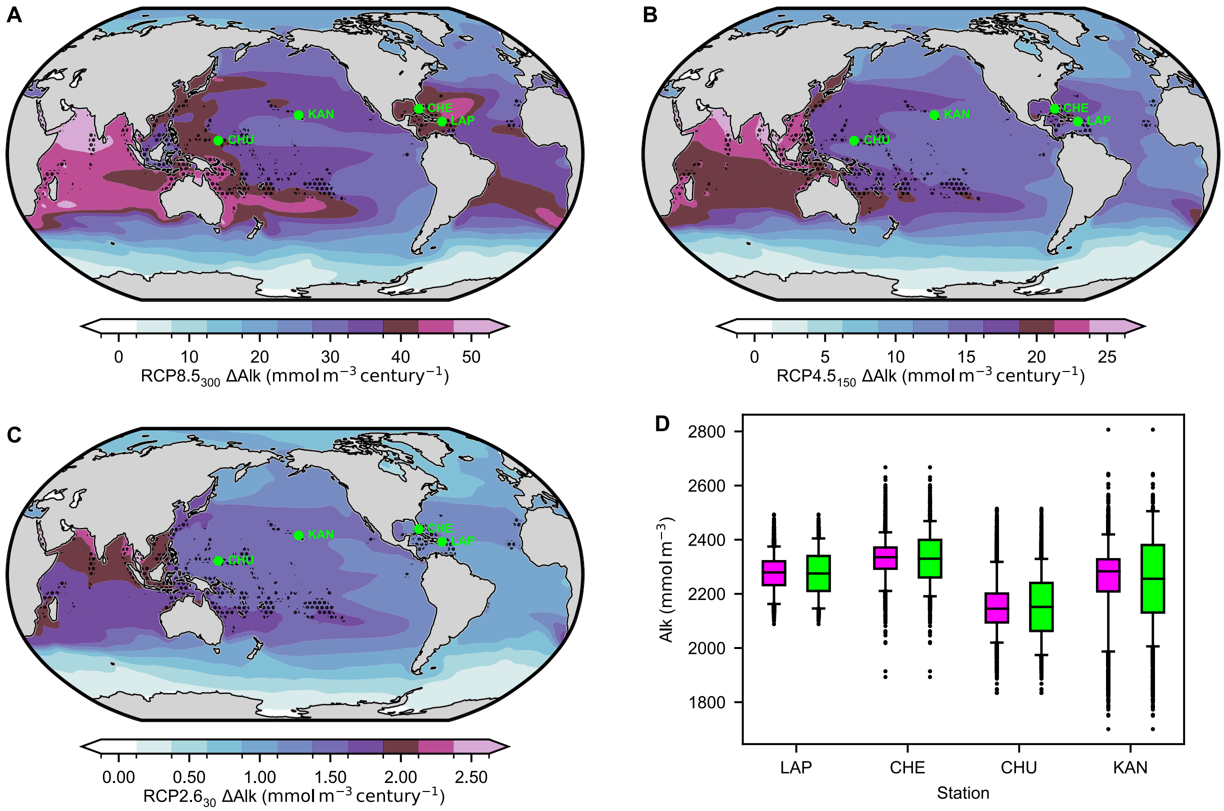

Fig S3. The potential detectability of trends in induced alkalinity anomalies. The simulated surface ocean trend in alkalinity due to coral reef carbonate production declines (ΔAlk; mmol m^{-3} $century^{-1}$) in (**A**), RCP8.5$_{300}$ (**B**), RCP4.5$_{150}$ and (**C**), RCP2.6$_{30}$, with stippling indicating the distribution of coral reefs. Alkalinity trends are relative to the respective RCP standard simulations (RCP8.5, RCP.4.5 and RCP2.6) and computed locally using linear regressions applied across the duration of the simulations. Regressions in all surface ocean grid cells are significant at the $p < 0.05$ level. Four coral reef time series stations of CO_2 system measurements (CHU: Chuuk Lagoon, KAN: Kaneohe Bay, CHE: Cheeca Rocks, LAP: La Parguera) are indicated on maps with (**D**), box and whisker plots of the alkalinity measurements at each station. Station alkalinity is recalculated with CO2SYS (2) using contemporaneous in situ pCO_2, pH, temperature and salinity measurements (3), and total dissolved inorganic phosphorus and total dissolved silicon concentrations taken from the nearest grid cell of the annual-mean World Ocean Atlas climatology (4). Purple box and whisker plots indicate the median (line), interquartile range (box), 5th and 95th percentiles (whiskers) and outliers (points). Green box and whisker plots indicate the mean (line), standard deviation (box), two times the standard deviation (whiskers) and outliers (points).

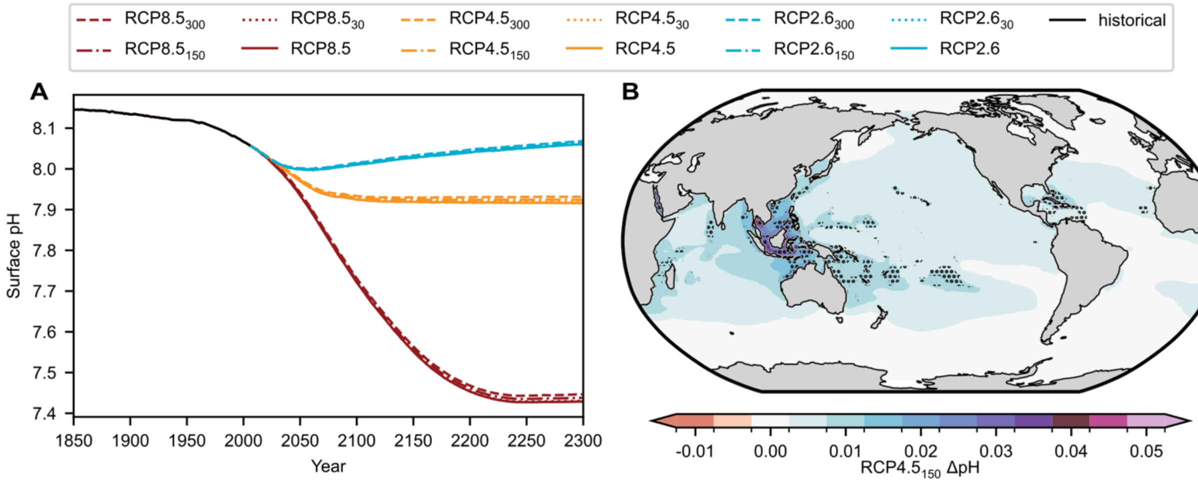

Fig S4. Declining coral reef carbonate production has limited influence on projections of ocean acidification. The projected (**A**), global mean surface ocean pH for all simulations and (**B**), the anomalies in surface ocean pH in 2090-2110 of $RCP4.5_{150}$ relative to RCP4.5. Stippling indicates the distribution of coral reefs.

SI References

1. A. R. Fay, *et al.*, SeaFlux: harmonization of air–sea CO_2 fluxes from surface pCO_2 data products using a standardized approach. *Earth System Science Data* **13**, 4693–4710 (2021).

2. S. van Heuven, D. Pierrot, J. Rae, E. Lewis, D. W. R. Wallace, CO2SYS v 1.1, MATLAB program developed for CO2 system calculations. *ORNL/CDIAC-105b. Carbon Dioxide Information Analysis Center, Oak Ridge National Laboratory, U.S. DoE, Oak Ridge, TN.* (2011).

3. A. J. Sutton, *et al.*, Autonomous seawater pCO_2 and pH time series from 40 surface buoys and the emergence of anthropogenic trends. *Earth System Science Data* **11**, 421–439 (2019).

4. H. Garcia, *et al.*, World Ocean Atlas 2018. Vol. 4: Dissolved Inorganic Nutrients (phosphate, nitrate and nitrate+nitrite, silicate). (2019).
